# Working memory and set-shifting in school-aged children classified as having attention-deficit hyperactivity disorder

**DOI:** 10.4102/sajpsychiatry.v28i0.1729

**Published:** 2022-01-28

**Authors:** Ramatladi M. Mphahlele, Anneke Meyer, Basil J. Pillay

**Affiliations:** 1 Department of Behavioural Medicine, Faculty of Health Sciences, University of KwaZulu-Natal, Durban, South Africa

**Keywords:** ADHD, age, digit backward, digit forward, gender, set-shifting, trail making-B, working memory

## Abstract

**Background:**

Attention-deficit hyperactivity disorder (ADHD) is a common psychiatric disorder reported in both children and adults; it is often associated with a variety of executive functioning deficits.

**Aim:**

This study investigated the extent to which working memory and set-shifting are impaired in school children with and without ADHD.

**Setting:**

This included primary schools in Lepelle-Nkumpi Municipality in Limpopo province, South Africa.

**Methods:**

A total of 216 children (108 screened positive for ADHD and 108 matched controls without ADHD symptoms), aged between 6 and 15 years, participated in the study. The performance of the two groups was compared on tests of working memory (Forward and Backward Digit Span subtests of the Wechsler Intelligence Scale for Children – Fourth Edition) and set-shifting (Trail Making Test Part B). The scores were analysed as a function of gender and age.

**Results:**

The group with possible ADHD performed worse than the neurotypical control group on tasks of working memory and set-shifting. The results did not indicate that gender affected performance. However, the younger age group performed worse than the older children.

**Conclusion:**

Children classified as ADHD showed significantly more impairments in working memory and set-shifting than neurotypical controls. Neither test showed any significant difference between male and female performance, whilst age was shown to affect performance on both tests. Early identification and treatment of children with attention-deficit hyperactivity disorder are crucial to their well-being.

## Introduction

Attention-deficit hyperactivity disorder (ADHD) is a debilitating neurodevelopmental disorder, characterised by extreme inattention and hyperactivity impulsiveness (HI), which interfere with daily functioning.^1^ It affects 5% of children and 2.5% of adults worldwide,^2^ and 7.5% of children and adolescents in African countries.^1^

The male-to-female ratio of ADHD in the general population is 2:1 in children and 1.6:1 in adults.^2^ The gender-skew could be attributed to the increased likelihood that female children display significantly more inattention, whilst male children show significantly more symptoms of combined HI and inattention.^3,4^ Thus, girls with ADHD tend to show fewer hyperactive and/or impulsive symptoms when compared with boys with ADHD.^2^ As a result, girls with ADHD are often not identified and are consequently under-represented in ADHD studies.

Some studies have shown more symptoms of hyperactivity in younger children, and more impulsiveness in adolescents.^2,4^ However, although younger children display more hyperactive or impulsive symptoms, the presentation shifts to more inattention as they grow older.^5^ The occurrence of age differences may be associated with delayed maturation of the post-central gyrus^6^ and increased ventral striatum activation in response to rewards.^7^ Studies, which have attempted to understand behaviour dynamics in ADHD, have shown an association between ADHD and executive functions (EF) deficits.^8,9^

*Executive functions* refer to a set of interconnected, higher order cognitive processes that control goal-directed behaviours and problem-solving.^10,11^ Executive functions can be classified into three main domains: working memory (WM), inhibitory control and set shifting.^10,12^ These facilitate goal-directed behaviour, planning^10,13^ and delay intolerance, which is the tendency to show a stronger preference for immediate smaller rewards over delayed larger rewards, in the case of simple choice tasks.^14^

Barkley’s EF model^15^ stipulates that although people with ADHD often report having a good memory, they experience difficulty with information retrieval when necessary, and struggle to keep track of other issues while attending to different tasks. Barkley’s model stresses the importance of self-directed behaviour to explain the difficulties of children with ADHD. According to this model, successful EF-related behaviour is regulated in the prefrontal cortex and requires the individual to use six self-directed actions: attention, restraint (e.g., inhibition), sensing (e.g., nonverbal WM), speech (verbal WM), emotions and motivations, and finally, planning, to adapt their behaviour and achieve a desired goal. Dysfunction of the prefrontal cortex results in HI, inattention and deficits in EFs.^16^ Given that EF difficulties are associated with ADHD, this study focused on testing individuals by means of the standardised performance-based measures of WM and set-shifting.

*Working memory* refers to systems that are necessary to enable the individual to remember information over the short-term and mentally use this information to learn, understand and reason.^11,17^ Baddeley^18^ proposed two basic subsystems for maintaining information – the visuospatial sketchpad that holds visual information and the phonological loop that stores auditory material. The WM model^19,20^ posits that children with ADHD tend to direct their attention to preferred activities, which place minimal demands on the central executive (CE) and the phonological short-term memory (PHSTM). It appears that children with ADHD pay attention to what they prefer, which means that they often display increased inattentiveness on tasks that require more WM function, when compared with neurotypical controls.^21^ Inattention in children with ADHD is largely related to their WM deficits,^21,22^ as an improvement in visual WM is associated with a decrease in symptoms of inattention.^23^

Working memory functions are located in the mid-lateral prefrontal cortex and the interconnected networks, which involve updating, dual processing and serial or temporal recording.^24^ A diversity of ADHD symptoms is caused by WM deficits,^13,25^ which may contribute to problems with inhibition and variable attention span, such as impulsiveness, and losing track of others’ conversations,^2^ as children with ADHD tend to display more WM deficits,^26^ which are not related to the limitations in the hippocampus.^23^

In terms of gender differences in WM, a meta-analysis of 11 studies by Maric et al.^27^ found increased WM deficits in boys with ADHD, a finding which was contradictory to that of Fried et al.,^26^ who showed that gender differences were not significant in WM. Similarly, the meta-analytic study by Savci et al.,^28^ aimed at reviewing the impact of EF in ADHD and its treatment, found no gender differences in WM functioning.

There have been mixed opinions as to whether age affects WM functions amongst children with ADHD. Maric et al.^27^ found age differences in children’s ability to sustain memory functions whilst engaging in cognitive activities, whereas Skogli et al.^29^ reported contrary findings. This suggests that although WM functions are partly determined by environmental factors, the underlying neuropsychological mechanisms remain the same across the different age categories.^8^

*Set-shifting* is ‘the ability to shift between tasks, to strategize diverse ways to resolve a problem, and to look at events from a different angle.’^30,31,32^ Children with ADHD exhibit difficulties in sustaining attention,^2^ and more set-shifting deficits, when compared to neurotypical controls.^33,34^ However, Irwin et al.^30^ showed that children with ADHD could sustain attention whilst switching between different domains as effectively as the control groups. This suggests that although EF deficits are more pronounced in ADHD, these deficits are not limited to children with ADHD,^26,28,33,34,35^ as the controls also showed deficits.^28^

Some studies^35,36^ demonstrated no gender differences in set-shifting as measured by performance in neuropsychological tests. It appears that set-shifting improves with age as the older children out-performed the younger ones, who required a longer time to complete set-shifting tasks.^28,36^ However, some studies^33,34,37^ suggest that age does not affect set-shifting performance from childhood to early adulthood, as adults with putative ADHD were also found to have impaired set-shifting abilities.^31^

The reported deficits in WM^13^ and set-shifting^33,34^ amongst children with ADHD appear to be neurologically based. Changes in the frontal lobe are implicated in EF deficits,^38^ which often lead to behavioural and emotional impairments, functional disturbances and academic failures.^39^ The frequently observed brain and behavioural abnormalities in ADHD may have genetic origins,^40^ which suggests that the structural deficits in brain networks require enabling genes and environment to produce functional impairments.^4,40^

The main aims of this study were to establish whether children with possible ADHD symptoms have deficits in WM and set-shifting as measured by the Digit Span and Trail-making Test Part B, respectively, and to examine their within-group gender and age differences in performance.

## Research method and design

### Study design

A case-controlled, quantitative, experimental design was employed to establish whether children who screened positive for ADHD are impaired in WM and set-shifting. The sample was divided into participants classified as ADHD and matched controls without symptoms of ADHD.

### Setting

Eleven primary schools from rural areas of the Limpopo province of South Africa were selected for the study.

### Study population and sampling strategy

Participants were Sepedi-speaking, Grade 1–7 learners, aged 6–15 years, whose teachers and parents completed the Disruptive Behaviour Disorders (DBD) rating scale^43,44^ to identify children with ADHD symptoms. A total of 5451 learners were screened using the DBD rating scale. Children with scores above the 95th percentile were selected as the ADHD group.^15,42^ Learners who scored below the 85th percentile formed the control group, in order to decrease the risk of false positives in the group. Children with scores of ≥ 26 on either or both the Inattentive and HI scales, matched for gender and age, were assigned to the ADHD group. These cut-off points were based on the results of the previous study by Meyer et al.,^41^ which was conducted on more than 6000 children in the Limpopo province and also employed the DBD. The control group, matched for gender and age, was established from the remaining cohort of children, who did not meet the inclusion criteria.

The resulting sample (*N* = 216) comprised 100 male and 116 female participants, and contained children who screened positive for ADHD symptoms (*n* = 108:50 males and 58 females) and a matched control group (*n* = 108:50 males and 58 females). Based on collateral information obtained from parents, teachers and school records, children with cerebral palsy, severe psychopathology, auditory and visual impairments, and intellectual disabilities were excluded from participation in the study.

### Data collection

This study was conducted in primary schools in the Lepelle-Nkumpi Municipality of the Capricorn district, Limpopo province, South Africa, during the year 2016. School principals provided consent for the study to be undertaken at their school. Informed, written consent was obtained from parents. The response rate for the DBD questionnaires was 96%. The testing procedure was explained to all learners selected for testing. The assessments were administered by clinical psychologists, with the help of field workers with psychology qualifications and who were conversant in Sepedi. Participants were individually assessed in a quiet, well-ventilated room. The test battery was administered during school hours, in accordance with the Department of Education’s regulations, in two sessions each lasting for approximately 60 min per participant. The total number of correct responses for the Digits subtest and the time taken to complete the Trail making test part B (TMT-B) were recorded.

#### Instruments

Disruptive Behaviour Disorders: The teachers screened the children for the presence of ADHD symptoms using the 18-item DBD rating scale^43,44^: inattention (9 items); HI (9 items). The parents also completed the DBD rating scale to confirm the existence of symptoms, as the symptoms must be present in two settings.^2,45^ The DBD was used to assess the presence and severity of ADHD symptoms; its 18 items diagnostic criteria are based on the fifth edition of the Diagnostic and Statistical Manual of Mental Disorders (DSM-5)^2^ as updated from the fourth edition of the DSM-IV-TR.^45,46^ The study employed the DBD as it is translated, normed and standardised for the Sepedi-speaking participants.^41^ Subsequent studies^47,48^ found the DBD to be a valid and reliable screening tool for ADHD amongst the Sepedi-speaking school children.

Each respondent was asked to rate the behaviour of the child on a four-point Likert scale: not at all (0), just a little (1), pretty much (2) and very much (3). Cronbach’s α for the targeted population was calculated at 0.92 for the inattention scale and 0.90 for the HI scale.^41^ For this study sample, Cronbach’s α was 0.74 for the HI scale and 0.79 for the inattention scale.

#### Digit span

Both Digits Forward and Digits Backward from the Wechsler Intelligence Scale for Children – Fourth Edition (WISC-IV)^49^ were administered to assess the phonological loop (forward digit span) and CE (backward digit span) components of WM.^11,18^ Digits Forward (FDS) involves the passive maintenance of verbal information, whereas the backward version (BDS) requires the active manipulation of that information. In the FDS, the participant was presented with an oral series of digits that they had to recall and repeat immediately in the same order, whereas for the BDS, the participants had to recall immediately but repeat the digits in the reverse order. The length of the trials increased; they began with a series of two digits, and one digit was added for each level. Each level contained nine trials of the same length, each divided into a series of three. A series was successfully achieved when the participant did not make any errors in at least two of the three trials. If the participant did not achieve a series, the task was discontinued. One point was awarded for each span level that was successfully achieved. Higher score indicates more effective WM functions. Adequate psychometric properties have been established for the subtest.^49^ Internal reliability ranges from 0.83 to 0.90, with construct validity of 0.1-0.5.^50^ The Cronbach’s α for this study was calculated at 0.78.

#### Trail making test part B

The TMT-B^51^ measures the participant’s ability to keep other matters in mind whilst attending to a task.^15^ The TMT-B consists of 25 circles which are distributed on a sheet of paper. The TMT requires the participant to connect a series of letters and numbers in ascending order by alternating between numbers and letters (number-letter switching: 1-A, 2-B, etc.).

Participants were instructed to work as quickly as possible, without lifting their pen or pencil from the paper. Whenever the child made an error, this was pointed out immediately, and they were allowed to self-correct. Errors affected the score as their correction increased the completion time of the task. Higher scores indicate difficulties in set-shifting.^34^ Test-retest reliability ranges from 0.60 to 0.90.^32^ The Cronbach’s α for the present sample was 0.67 and 0.72 for Part A and B, respectively.

### Data analysis

Statistica 12.7 (Dell, 2015)^52^ was used for statistical analysis. MANOVA models were employed to examine probability between-group differences. *Between-group differences* were *established using a* 2 × 2 × 2 (ADHD group × gender × age) multifactorial ANOVA. Post hoc analysis (Bonferroni) was used to determine within-group differences (differences in gender and age).

### Ethical considerations

Ethical clearance was obtained from the Biomedical Research Ethics Committee of University of KwaZulu-Natal, Durban, South Africa (reference number HSS/1446/015D) and the Department of Education, Limpopo province, South Africa. Principals of the selected schools provided their verbal consent for the study to be undertaken in their schools. Written, informed consent was obtained from parents or legal guardians of the learners to participate in the study. The researchers read out the assent form to children, and, after it was established that they had understood and agreed with the content, they were asked to assent to take part in the study. All children irrespective of age were treated in the same way. Voluntary participation was ensured, as participants were informed that they could withdraw at any stage should they want to do so. Parents of children who obtained positive scores were referred to the nearest hospital for further assessments and management.

## Results

### Digit span

Descriptive statistics, the MANOVA and the post hoc results for Digit Span are shown in Table 1. No main or interactions effect was found for gender. Statistical differences were found in performance between the ADHD and control group: Wilks L = 0.65, *F* = 56.15, *p* < 0.001, *ƞ*p^2^ = 0.35. A main effect for age was indicated: Wilks L = 0.90, *F* = 11.48, *p* < 0.001, *ƞ*p^2^ = 0.10, whilst no interactions effect was revealed for age. Post hoc analysis (Bonferroni) revealed that for both the Digits Forward and Digits Backwards, the older age group performed significantly better than the younger age group (both *p* < 0.001). An effect size (partial eta squared, *ƞ*p^2^) of 0.35 (ADHD vs. control) can be considered as large and of 0.10 (difference in age) as medium.^53^

**TABLE 1:** Descriptive statistics and MANOVA results for digits.

Traits	ADHD				Control				Group comparison DF (1, 208)				Post hoc	Bonferroni *p*
*N*	%	*M*	SD	*N*	%	*M*	SD	Wilks L	*F*	*p*	*ƞp^2^*			
Group	-	-	-	-	-	-	-	-	0.65	56.15	< 0.001*	0.35	-	-
Gender	-	-	-	-	-	-	-	-	0.99	0.44	0.65	0.00	-	-
Age	-	-	-	-	-	-	-	-	0.90	11.48	< 0.001*	0.10	-	-
Group × gender	-	-	-	-	-	-	-	-	0.99	0.73	0.48	0.01	-	-
Group × age	-	-	-	-	-	-	-	-	0.10	0.01	0.99	0.00	-	-
Gender ×age	-	-	-	-	-	-	-	-	0.98	0.82	0.49	0.01	-	-
Group × gender × age	-	-	-	-	-	-	-	-	0.99	1.61	0.20	0.02	-	-
**FDS** 6–10 11–15	66 42	30.6 19.4	6.49 7.62	1.77 1.82	66 42	30.6 19.4	7.91 8.95	1.62 1.77	- -	- -	- -	- -	6 – 10 versus 11–15 age group	< 0.001
**BDS** 6–10 11–15	66 42	30.6 19.4	1.64 2.40	1.27 1.21	66 42	30.6 19.4	3.82 4.55	1.71 1.60	- -	- -	- -	- -	6–10 versus 11–15 age group	< 0.001

ADHD, attention-deficit hyperactivity disorder; TMT-B, trail making test – part B.

### Trails-B

Descriptive statistics, the ANOVA and the post hoc results for Trails-B are shown in Table 2. No main or interactions effect was indicated for gender. Statistical differences were found in performance between the ADHD and control group: *F* = 69.74, *p* < 0.001, *ƞ*p^2^ = 0.25. A main effect was indicated for age: *F* = 49.67, *p* < 0.001, *ƞ*p^2^ = 0.19, whilst no interactions effect for age was revealed. Post hoc analysis revealed that the older group performed significantly better than the younger age group (*p* < 0.001). Effect sizes (partial eta squared, *ƞ*p^2^) of 0.25 (ADHD vs. control) and of 0.19 (difference in age) can be considered as large.^53^

**TABLE 2:** Descriptive statistics and ANOVA results for trails–B.

Traits	ADHD				Control				Group comparison DF (1, 208)			Post hoc	Bonferroni *p*
	*N*	%	*M*	SD	*N*	%	*M*	SD	*F*	*p*	*p* ^2^		
Group	-	-	-	-	-	-	-	-	69.74	< 0.001	0.25	-	-
Gender	-	-	-	-	-	-	-	-	0.90	0.34	0.00a	-	-
Age	-	-	-	-	-	-	-	-	49.67	< 0.001	0.19	-	-
Group × gender	-	-	-	-	-	-	-	-	10.20	0.002	0.05	-	-
Group × age	-	-	-	-	-	-	-	-	6.56	0.01	0.03	-	-
Gender ×age	-	-	-	-	-	-	-	-	1.16	0.28	0.01	-	-
Group × gender × age	-	-	-	-	-	-	-	-	0.02	0.90	0.00	-	-
**TMT-B** 6–10 11–15	66 42	30.6 19.4	383.32 247.57	131.69 121.95	66 42	30.6 19.4	222.46 156.62	91.19 51.30	- -	- -	- -	6 – 10 versus 11–15 age group	< 0.001

ADHD, attention-deficit hyperactivity disorder; TMT-B, trail making test – part B.

## Discussion

This study investigated whether gender and age differences in performance in children with and without possible ADHD may be because of the specific components of the WM system, namely, the active versus passive processing, or the coding, namely, the visuo-spatial or verbal coding, and whether the ability to keep other details in mind whilst attending to other tasks (set-shifting) may be affected by gender and age. In order to achieve these aims, two standard instruments were employed, namely, the two processing conditions of the DS (i.e. forward and backward repetition) that measure passive and active components of WM, respectively,^49^ and the TMT-B that assesses set-shifting.^51^ Thus, this study investigated set-shifting by studying the ability of participants to alternate between numbers and letters.

It was found that children who screened positive for ADHD symptomatology performed more poorly than the controls in respect of both WM and set-shifting. Gender did not affect their performance on either the DS or TMT-B; however, specific age differences were detected by both tests.

### Working memory deficits in attention-deficit hyperactivity disorder

The results reveal that children with symptoms of ADHD, when compared with controls, showed significantly more WM deficits. This finding is in accordance with those of previous studies,^54,55^ which suggested a significant difference between the two groups. The finding of this study supports Barkley’s EF model,^15^ which posits that children with putative ADHD experience difficulty retrieving information and keeping other details in mind whilst they attend to other activities. A study by Elosúa et al.^32^ also showed more WM deficits in children with possible ADHD, as indicated by lower scores on both the Digits Forward and Backward tests, when compared with the controls.

This study showed no gender differences in WM deficits in either the group with possible ADHD or the neurotypical controls. This finding contradicts previous findings by Skogli et al.^56^ which showed higher rates of WM deficits in females with putative ADHD. The absence of gender interaction suggests that group differences in WM deficits may not be due to gender, as has previously been reported, where boys showed significant WM deficits when compared to girls.^27^ This finding echoes some previous studies^26,28^ that found no statistically significant gender differences in WM deficits amongst children with possible ADHD. A study by Piccardi et al.^57^ also found no gender differences in WM amongst young adults (21–35 year olds) on DS, which suggests that in the case of persons who screened positive for ADHD, at least from childhood through to young adulthood, gender does not affect their ability to remember information over a short period of time, or to mentally utilise such information to learn, comprehend, and reason.

The findings of this study indicate the group independent age differences in WM functions, by showing that older children outperformed their younger peers on the Digits Span measurements, irrespective of whether they have possible symptoms of ADHD. This finding is contrary to some studies, which found that age did not affect the ability of children (viz. possible ADHD and controls) to sustain memory functions whilst engaging in cognitive activities.^26,27,34^ The finding that younger children with or without putative ADHD displayed more deficits in WM suggests that the WM functions have possible neurodevelopmental basis associated with the maturation of the frontal lobe. Although it was not the primary focus of the current study, the fact that we could not find the group-related age differences in performance on functions of WM offers an explanation to previous research,^13,24^ which reported that frontal lobe matures with age; however, the maturation does not depend on whether the person screened positive for ADHD symptoms. This finding is consistent with some previous studies,^23,58^ which found that age significantly improved WM functioning in children with possible ADHD with regard to computerised tasks. In terms of age differences in WM functions amongst children with ADHD, the meta-analytical study by Kasper et al.^25^ showed that WM functions tend to improve with age amongst children with possible ADHD and typically developing children aged 8–16 years, which suggests that although these functions are partly determined by environmental^22^ influences, the underlying developmental factors^7,59^ and brain alterations that influence WM functioning^24^ are similar across groups (viz. possible ADHD and Controls). This finding echoed that of Karalunas et al.,^23^ which suggested that individual differences in visuospatial WM during the late childhood (7 through 13 years of age) predict ADHD symptom improvement as those who performed better in WM tasks also showed adequate improvement in ADHD symptoms. Consequently, older children with possible ADHD would be expected to show improved functioning, as they would have more time to developmentally ‘catch up’ to their typically developing peers.

### Set-shifting in attention-deficit hyperactivity disorder

Consistent with some previous studies,^32,34^ this study found that children with possible ADHD performed poorly and made more errors on the TMT-B, and therefore, often required more time to complete the test when compared with the controls. This suggests that children with putative ADHD exhibit more deficits in set-shifting skills.^33,34^ However, some studies found that children who screened positive for ADHD effectively sustain attention whilst switching between different domains.^28,30^ Poor performance on the TMT indicates brain dysfunction in the prefrontal cortex, which is responsible for the set-shifting functions.^30^

This study found no gender differences in set-shifting performance, which suggests that although behaviour is influenced by environment or culture, the neuropsychological mechanisms underlying behaviour are the same across genders.^8,24^ This finding converges with that of other studies,^28,35^ which reported that children who screened positive for ADHD, irrespective of gender, performed poorly on the TMT-B, and therefore, took more time to complete the task. Consistent with some previous studies,^28,26^ older children performed better than the younger age group, who needed more time to complete the TMT-B. This finding departs from that of Rose et al.^37^ who found no age differences in children’s ability to keep other matters in mind whilst attending to tasks.

Overall, these findings suggest that children with putative ADHD displayed more EF deficits, which supports Barkley’s model^21^ that children with possible ADHD have difficulty retrieving information from memory and keeping other details in mind whilst attending to different tasks.^15^ However, Minervino and Pereira^60^ found non-significant group differences in WM and set-shifting, which suggests that individual deficits may not explain the multifaceted nature of ADHD. The contradictory results may imply that other psychological processes, which were not controlled in this study, may be involved. Whilst neuroimaging was not conducted, the results would be theoretically consistent with deficiencies in the dopamine reward valuation system.^38^ In line with Seidman et al.,^8^ this study did not find gender differences in executive functioning, which suggests that whilst diverse environmental and/or cultural factors have impact on behaviour, possibly similar neuropsychological mechanisms underlie the executive functioning in children with putative ADHD across both genders. The fact that older children performed better than the younger age group could imply that ADHD and executive functioning are developmental processes.^28^ In addition, the age of the sample may account for the lack of gender differences, as the gender-related differences may only be detected from the age of 12 years, with the onset of puberty,^59^ when pubertal gonadal changes and related increased neural activation in the ventral striatum play a pivotal role in heightening ADHD symptoms.^7^ The ventral striatum is a part of the brain’s reward system that increases in typically developing pubertal samples, following positive reward, and is associated with heightened gonadal hormones.^7^

The implications of this study are that children with putative ADHD symptoms are significantly impaired in WM and set-shifting skills. The literature suggests that children with possible ADHD, and with deficits in WM^26^ and set-shifting acuity,^33,35^ often perform poorly at school when compared with the typically developing control group.^2,4^ Earlier identification and intervention for children screening positive for ADHD and EF deficits can improve the functioning of these children.

## Limitations

Firstly, the homogeneity of the sample, as our mean age was 10 years, limited a significant representation for adolescents. Secondly, the sample only represented Sepedi-speaking children from one specific area in Limpopo, and therefore, cannot be generalised. For further investigation, heterogeneous samples, where different measurements are used, should be considered.

## Conclusion

The current study showed that children with possible ADHD symptomatology display more deficits in WM and set-shifting when compared with controls without putative ADHD. Amongst children classified as ADHD, no gender differences were found in their performance on the selected tasks of EFs, whilst the younger children appeared to be significantly impaired on both WM and set-shifting, compared with the older ones. Children with possible ADHD and executive dysfunction are more likely to be significantly impaired in both academic and social functioning, given their poorer attention, concentration, memory and impulsiveness^2^.

This debilitating effect of executive dysfunction in children with putative ADHD has been demonstrated in the literature,^2,4^ which has clinical implications for the management and treatment of these children.
